# A Miniature Four-Channel Ion Trap Array Based on Non-silicon MEMS Technology

**DOI:** 10.3390/mi12070831

**Published:** 2021-07-16

**Authors:** Qi Zhang, Xichi Lu, Ting Chen, Yu Xiao, Rujiao Yao, Jinyuan Yao

**Affiliations:** 1 National Key Laboratory of Science and Technology on Micro/Nano Fabrication, Shanghai Jiao Tong University, Shanghai 200240, China; qzhang31@sjtu.edu.cn (Q.Z.); xichi.lu@sjtu.edu.cn (X.L.); 17606081988@163.com (T.C.); 2 Department of Micro/Nano Electronics, School of Electronic Information and Electrical Engineering, Shanghai Jiao Tong University, Shanghai 200240, China; 3 Institute of Spacecraft Equipment, Shanghai 200240, China; xiaoyush812@126.com (Y.X.); rjsh812@126.com (R.Y.)

**Keywords:** miniature four-channel ion trap array (MFITA), MEMS, mass spectrometry

## Abstract

With the increasing application field, a higher requirement is put forward for the mass spectrometer. The reduction in size will inevitably cause a loss of precision; therefore, it is necessary to develop a high-performance miniature mass spectrometer. Based on the researches of rectangular ion trap, the relationship between mass resolution and structural parameters of the ion trap array was analyzed by further simulation. The results indicate that, considering the balance of mass resolution and extraction efficiency, the preferable values for the field radius of exit direction *y*_0_ and ion exit slot width *s*_0_ are 1.61 mm and 200 μm, respectively. Afterwards, a miniature four-channel ion trap array (MFITA) was fabricated, by using MEMS and laser etching technology, and mass spectrometry experiments were carried out to demonstrate its performance. The mass resolution of butyl diacetate with *m*/*z* = 230 can reach 324. In addition, the consistency of four channels is verified within the error tolerance, by analyzing air samples. Our work can prove the correctness of the structural design and the feasibility of MEMS preparation for MFITA, which will bring meaningful guidance for its future development and optimization.

## 1. Introduction

Mass spectrometry is an important material analysis method nowadays. It has been widely used in many research fields, such as chemical analysis, drug metabolism, clinical medicine, environmental protection, food safety, anti-terrorism, public emergencies, and military technology [1,2,3,4,5]. Although the conventional desktop mass spectrometer has a relatively high technical maturity, it has the disadvantages of large size, high power consumption, and high price, which cannot meet the growing demand. A miniature mass spectrometer can provide effective methods and means for mobile detection and on-site detection, and they are the main direction of mass spectrometry technology in the future. In particular, the handheld mass spectrometer is popular because of its extremely high portability and practicality. Yang et al. reported a handheld mass spectrometer PPMS that can be carried by individual soldiers for chemical warfare agent detection [6]. The MX908 handheld mass spectrometer, developed by the American device company, can be used in the fields of space exploration, emergencies, and public safety.

The miniaturization of mass spectrometers often starts with the miniaturization of mass analyzers [7]. In recent years, almost all mass analyzers are developing towards miniaturization [8], including quadrupoles, ion traps, etc. In particular, the ion trap mass analyzer has a simple structure, high sensitivity, low vacuum requirements, and can realize multi-stage tandem mass spectrometry [9], which has unique advantages in miniaturization. Traditional ion trap mass analyzers mainly include three-dimensional ion trap (Paul trap) and linear ion trap (LIT) [10,11]. Their electrodes adopt a hyperboloid or hyperboloid cylindrical structure, which both require high machining accuracy and increases the costs of production and processing [12]. Therefore, various ion traps with simplified electrode structures have gradually appeared. The cylindrical ion trap (CIT) proposed by Cooks et al. simplified the three hyperboloid electrodes of the three-dimensional ion trap into cylindrical ring electrodes and two parallel end cap electrodes, which greatly reduced the processing difficulty [13]. Based on CIT, a rectangular ion trap (RIT) is further proposed, which is surrounded by only six planar electrodes, and combines the simple structure of CIT with the large storage capacity and high trapping efficiency of LIT [14]. Jiang et al. proposed a PCB-based rectangular ion trap mass analyzer (PCB ion trap), which uses ordinary PCB materials and processing technology to manufacture ion traps with simpler structure and low price [15]. Li et al. reported a PCB ion trap array based on the PCB ion trap, which contains multiple ion analysis channels, which greatly improves the analysis throughput of the ion trap [16].

However, in the miniaturization process of the ion trap structure, as the size decreases, the analyte storage capacity of a single ion trap channel will also decrease, which causes great attenuation of the ion trapping efficiency and analysis throughput. Furthermore, if the concentration of the analyte exceeds the limit storage capacity of the ion trap, a serious space charge effect will occur inside the ion trap channels, which will reduce the mass resolution. Well et al. reported that the mass spectrometry of n-butylbenzene was analyzed by CIT, with a field radius of 1 cm, and the mass resolution was 300 [17]. Badman et al. fabricated a CIT array with a field radius of 2.5 cm, to increase the ion storage capacity, and the mass resolution obtained by analyzing M-dichlorobenzene was 180 [18]. Maas et al. reported a RIT array with a field radius of 1.33 mm, and the mass resolution was no more than 40 [19]. In addition, the analytical performance of the ion trap also depends on the accuracy of electrode processing and manufacturing [20]. Compared with conventional micromachining methods, MEMS technology has gradually become the mainstream for the fabrication of miniature ion traps, because of its high precision. Blain et al. used silicon-based MEMS and tungsten metallization technology to process 106 CIT in an area of 0.25 cm^2^ on a chip, with a field radius of 1.0 μm for each CIT [21]. Malcolm et al. reported a MEMS-based miniature mass spectrometer Microsaic 3500MiD, with a mass range of up to 800 amu [22]. Huang et al. optimized the MEMS-based RIT structure with a field radius of 400 μm, and the RF voltage parameters, and successfully achieved ion trapping [23].

Miniature ion traps are mainly based on metal, and its fabrication involves process compatibility issues of non-silicon material. Therefore, there are few experimental researches on miniature ion traps based on non-silicon MEMS technology. Most of the related researches only focus on the simulation, and fail to combine the simulation results with the actual fabrication, and complete the actual test. Moreover, the existing related researches mainly focus on parameters such as ion capture efficiency and storage rate, and there are few discussions on key performance parameters such as mass resolution.

In this work, we designed the structure of the MFITA, which can compensate for the throughput loss caused by the miniaturization and improve the efficiency of mass analysis. Utilizing the software, including SIMION, AXISM [9,24] etc., the relationship between the mass resolution and structural parameters of the ion trap array was simulated. Considering the balance of mass resolution and extraction efficiency, the preferable values for the field radius of exit direction *y*_0_ and ion exit slot width *s*_0_ are obtained, that is, 1.61 mm and 200 μm, respectively. Afterwards, an MFITA was fabricated by using MEMS and laser etching technology, and mass spectrometry experiments were carried out to demonstrate its performance. The mass resolution of butyl diacetate, with *m*/*z* = 230, can reach 324. In addition, the consistency of four channels is verified within the error tolerance by analyzing air samples. Our work can prove the correctness of the structural design and the feasibility of MEMS preparation for MFITA, which will bring meaningful guidance for its future development and optimization.

## 2. Materials and Methods

### 2.1. Structure and Fabrication

**Figure 1:**
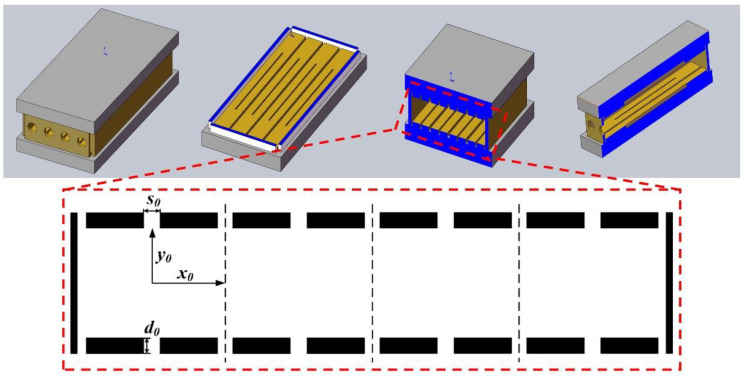
Structure diagram of MFITA.

Figure 1 is the structure diagram of the MFITA. Define the ion exit direction as the *y* direction, the field radius of the *y* direction is *y*_0_, the non-exit direction is the *x* direction, and the field radius is *x*_0_. The electrode thickness is *d*_0_. An ion exit slot is opened on the ion exit direction for ion ejection, and the width is *s*_0_.

**Figure 2:**
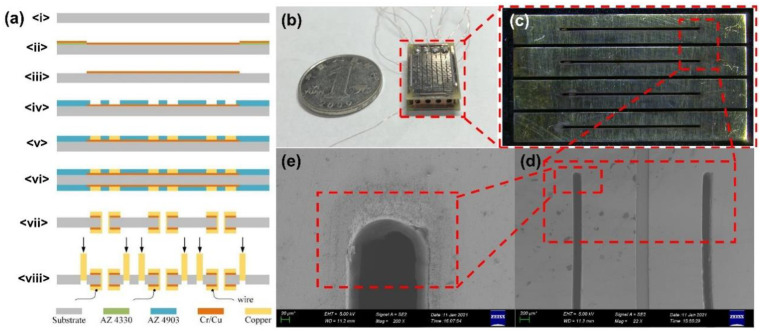
(**a**) Fabrication flow of MFITA. (**b**) A physical image of fabricated MFITA. (**c**) A top-view optical image of the MFITA. (**d**) A SEM image of ion exit slot. (**e**) Enlarged details for (**d**).

The two electrode plates in the *x* direction of the ion trap array are prepared by micromachining technology to ensure the high accuracy of the electrodes; the electrodes and end cap electrodes in the *y* direction are prepared by laser etching of copper sheets. The main process are described as follows (Figure 2a):

<i> Choose a three-inch FR-4 board substrate and clean it;
<ii> Use AZ 4330 photoresist on the substrate to homogenize and pattern, and then sputter Cr/Cu as the electroplating seed layer;
<iii> Use the lift-off process to remove the photoresist, leaving the Cr/Cu seed layer;
<iv> Use AZ 4903 photoresist to homogenize and pattern;
<v> Electroplate 50 μm copper as an electrode on the seed layer;
<vi> Use backside alignment lithography technology, repeat b–e steps on the backside of the substrate to fabricate backside electrodes;
<vii> Use wet etching process to remove the photoresist and seed layer, leaving the electrode, and sanding the electrode with water to ensure uniformity of surface;
<viii> Use FP/FPS ultraviolet micro-processing system and IGE glass fiber laser high-speed equipment to cut out ion exit slots and connect wires. Use MicroVector ultraviolet laser FPC cutting machine to cut the copper sheet as the *y*-direction electrode and end cap electrode, insert the electrode slot to assemble the ion trap array.

Laser etching is used to completely cut the y-direction electrode and the end cap electrode on a 500 μm copper plate, which can ensure the dimensional accuracy of the electrode while avoiding electrode bending. For the assembly of the ion trap, a principle similar to the mortise and tenon structure is adopted, and four copper electrodes are accurately positioned by cutting slots on the substrate with a laser device. The length of the left and right slots is 14 mm, and the length of the front and rear slots is 2 mm. The slots are long enough to accurately position four copper electrode plates on the upper and lower electrode plates. The slot penetrates the substrate, after the complete assemble, the four copper electrodes restrict each other in four directions, ensuring verticality and accuracy.

The fabricated miniature four-channel ion trap array is shown in Figure 2b. The field radius *x*_0_ is 1.61 mm, *y*_0_ is 1.40 mm, the electrode thickness *d*_0_ is 25 μm, the slot width *s*_0_ is 200 μm. The detailed optical and SEM images are shown in Figure 2c–e.

### 2.2. Configuration of Simulation

A gem file is written by SIMION software to define the electrode structure of the ion trap array and the voltage application method. A pair of balanced digital bound square wave voltages (equal amplitude and 180° phase difference) are applied to adjacent electrode pairs in the *x* direction (Supplementary Figure S1a). Therefore, a zero potential energy surface similar to a “virtual electrode” is formed between adjacent channels, which separates four channels to form independent electric field regions. A digital excitation square wave voltage is also applied to the upper and lower electrodes in the *x* direction and the electrodes of adjacent channels have opposite phases, which are used for the resonance excitation and ejection of ions (Supplementary Figure S1b). The electrodes in the *y* direction are grounded, so that the electric fields of the ion traps on both sides and the ion trap in the middle remain the same. The shape of the electric field in each channel is the same, similar to an independent miniature ion trap, which can work independently.

AXSIM software is used to set the initial conditions of ions and the force of ions at a certain time to obtain their trajectory, thereby obtaining the mass spectrum peak of the ion trap array and analyzing its performance. Put 300 ions with *m*/*z* of 117, 119, 121 in the ion storage analysis area of the ion trap array. The initial positions of the ions are randomly distributed in the range of 0–10 μm near the center of the ion trap. The cooling gas is helium, the background pressure is 0.13 Pa, the temperature is 300 K, and the collision model is hard ball collision model. In the simulation process, the “analog RF scanning” method is selected [25]. The RF signal adopts a sinusoidal signal with a frequency of 3.072 MHz, and the mass resolution is obtained by scanning the RF amplitude. There are the following two ion excitation methods: “boundary excitation” and “resonance excitation” [26]. In order to improve the mass resolution of the ion trap, the “resonance excitation” method is selected. The resonance excitation voltage AC is also a sinusoidal signal, the frequency is the third-frequency division of the RF voltage, and the amplitude is the minimum value that can make all ions eject from the ion trap. When the frequency of AC is the same as the characteristic frequency of ion motion, the ions will resonate, the amplitude of motion will increase sharply, and the ion trap will be ejected from the slot.

Theoretically, the ideal internal electric field can only be realized in the 3D ion trap that only contains ideal quadrupole field. The condition for obtaining the ideal quadrupole field is the space enclosed by four hyperboloid electrodes with infinite length. Since the miniature ion trap array adopts a simplified planar electrode structure, a certain proportion of high-order field components will be introduced. Inappropriate high-order fields may directly cause the loss of the mass analysis ability of miniature ion trap array. Therefore, it is necessary to study the electric field inside the ion trap array with different structures. The internal potential can be expressed as [14].

(1) Φ(x,y,t)=VRF×Re∑n=0∞An(x+iyr0)n×cos(Ωt)

where *V_RF_* and *Ω* are the amplitude and frequency of the RF voltage, *A_n_* (*n* = 0, 1, 2, 3,…) is the multipole field parameters, corresponding to DC electric field, dipole field, quadrupole field, hexapole field, etc., and *r*_0_ is the field radius. PAN33 software is used to obtain the multipole field components inside the ion trap array.

IC5Filter software is used to integrate the ion motion trajectories obtained by the AXSIM software, and draw the corresponding mass spectrum (Supplementary Figure S2). The mass resolution calculated by the unimodal method can be expressed as follows:

(2) $$R=\frac{M}{\Delta m}$$

where *Δm* is full width at half maximum (FWHM).

## 3. Results and Discussion

### 3.1. Parameter Optimization

**Figure 3:**
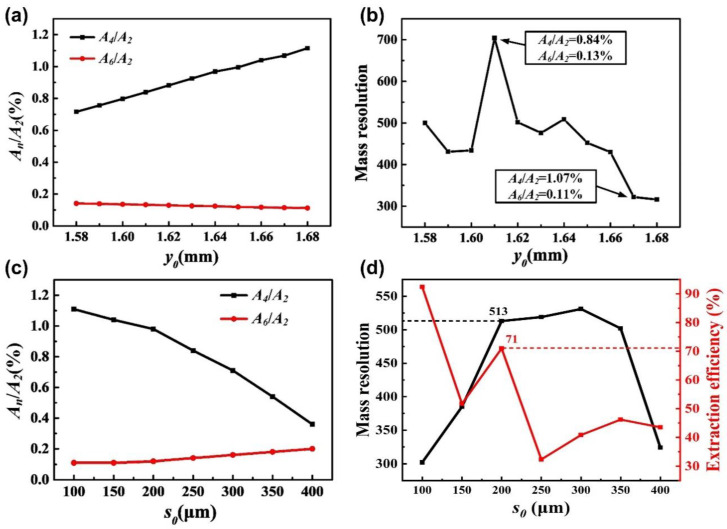
(**a**) Change in the internal electric field composition based on different *y*_0_. (**b**) Change in the mass resolution based on different *y*_0_. (**c**) Change in the internal electric field composition based on different *s*_0_. (**d**) Changes in the mass resolution and ion extraction efficiency based on different *s*_0_.

The field radius is one of the key factors that determines the performance of mass analysis [14,27], especially for the miniature ion trap. In order to obtain the optimal value of the field radius of exit direction *y*_0_, we simulated the effect of different *y*_0_ on the mass analysis performance of the miniature four-channel ion trap array. The given scanning speed is 2530 Th/s, the electrode thickness *d*_0_ is 25 μm, and the field radius of the non-exit direction *x*_0_ is 1.4 mm. We changed the range of *y*_0_ from 1.58 to 1.68 mm, and obtained one set of data every 10 μm, with 11 sets in total. Figure 3a shows the change in the internal electric field composition based on different *y*_0_. Each channel is an axisymmetric structure in the *x* and *y* directions, so the high-order electric field component in the ion trap array is only the dipole field *A_2n_* (*n* = 1, 2, 3, …), that is, the quadrupole field, the octopole field, and the twelve-pole field, etc. With the increase in *y*_0_, the value of the octopole field (*A*_4_/*A*_2_) shows an obvious linear increase trend, while the value of the twelve-pole field (*A*_6_/*A*_2_) shows an unobvious linear decrease trend, and gradually tend to 0.1%. In contrast, the octopole field accounts for more, and changes more significantly. The higher dipole field accounts for less, with changes so insignificant that they will not be discussed again. Figure 3b shows the change in the mass resolution based on different *y*_0_. When *y*_0_ is 1.61 mm, the miniature ion trap array reaches the best mass resolution of 704, the value of *A*_4_/*A*_2_ is 0.84% and the value of *A*_6_/*A*_2_ is 0.13%. When *y*_0_ is 1.67 mm, the value of the mass resolution drops to about 300, the value of *A*_4_/*A*_2_ is 1.07%, and the value of *A*_6_/*A*_2_ is 0.11%. It can be seen that the appropriate proportion of the octopole field component helps ion excitation, thereby obtaining a higher mass resolution, which is the same as the results of previous studies [28,29]. However, if the proportion is too large, the internal electric field composition will be unbalanced, which will damage the performance of the mass analysis.

For the emitting of ions, an ion exit slot is inevitably opened on the electrode, which causes the distortion of the electric field near the slot, thereby affecting the performance of the mass analysis. Therefore, we also simulated the effect of different ion exit slot widths *s*_0_ on the performance of the MFITA. The given *x*_0_ is 1.4 mm, *y*_0_ is 1.61 mm, and *d*_0_ is 50 μm. We changed the range of *s*_0_ from 100 to 400 μm, and obtained one set of data every 50 μm, with seven sets in total. Figure 3c shows the change in the internal electric field composition based on different *s*_0_. The octopole field accounts for a relatively high proportion, and gradually decreases exponentially with the increase in *s*_0_, while the twelve-pole field gradually increases with the increase in *s*_0_. When *s*_0_ reaches 400 μm, the difference between *A*_4_/*A*_2_ and *A*_6_/*A*_2_ in the ion trap array is less than 0.2%. At this time, the effect of the twelve-pole field on the internal electric field cannot be ignored. Figure 3d shows the changes in the mass resolution and ion extraction efficiency, based on different *s*_0_. When *s*_0_ is 100 μm, the extraction efficiency reaches the best, but the mass resolution is low. On the contrary, when *s*_0_ is 300 μm, to reach the best mass resolution, the extraction efficiency is low. If the changing trends of the mass resolution and extraction efficiency are quite different, the mass resolution should be given priority. Therefore, 200 μm was selected as the ion exit slot width of the miniature ion trap array. The mass resolution is 513, and the extraction efficiency is 71%, both of which have adequate values.

### 3.2. Mass Spectrometry Experiment

Through simulation, the optimal parameters, including the field radius of exit direction *y*_0_ and the ion exit slot width *s*_0_, were obtained; afterwards, our designed MFITA was fabricated by MEMS technology, and mass spectrometry experiments were carried out. The test platform used in our experiments is the self-built electron impact ion source (EI) mass spectrometer system (Supplementary Figure S3), and its detailed components and mechanism are discussed in the supporting data (Supplementary Note 1). The reagents that were used in our experiments were butyl diacetate (*m*/*z* = 230), with a purity of 99%, which were purchased from Shanghai ANPEL Laboratory Technologies Inc. The air samples were collected from the laboratory environment in the same batch.

**Figure 4:**
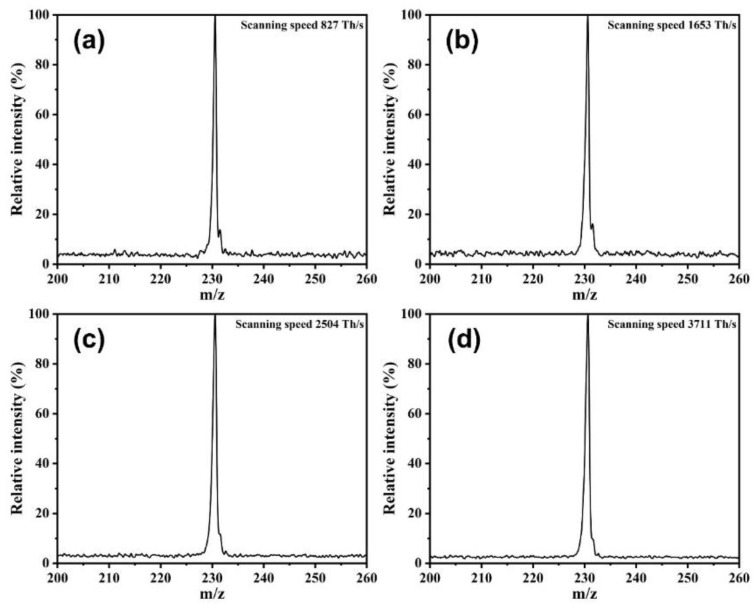
(**a**) Mass spectrum of butyl diacetate at scanning speed of 827 Th/s. (**b**) Mass spectrum of butyl diacetate at scanning speed of 1653 Th/s. (**c**) Mass spectrum of butyl diacetate at scanning speed of 2504 Th/s. (**d**) Mass spectrum of butyl diacetate at scanning speed of 3711 Th/s.

The mass resolution of our MFITA, based on different scanning speeds, was tested by using a butyl diacetate sample. Figure 4a–d show the mass spectrums of butyl diacetate at different scanning speeds. When the scanning speed is 827 Th/s, the value of FWHM is 0.71, and the corresponding value of mass resolution is 324. With the increase in the scanning speed, the value of FWHM at signal peak gradually increases, which leads to the decrease in the mass resolution.

**Figure 5:**
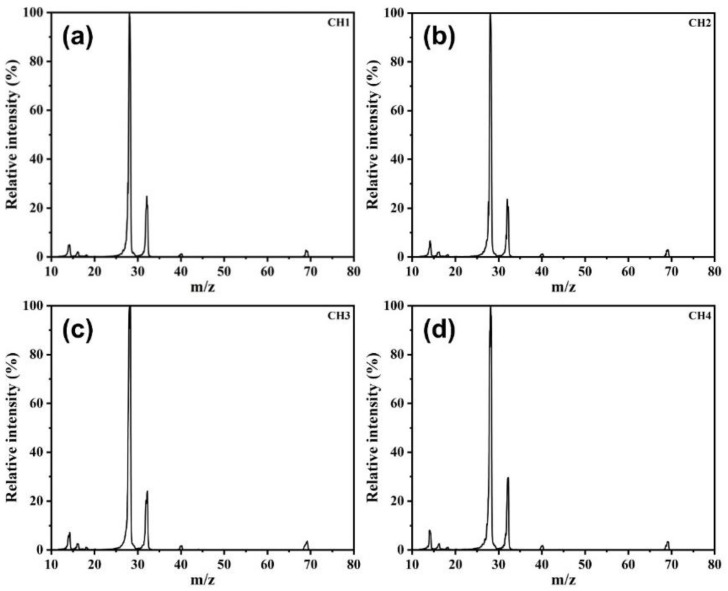
(**a**) Mass spectrum of air sample in channel 1. (**b**) Mass spectrum of air sample in channel 2. (**c**) Mass spectrum of air sample in channel 3. (**d**) Mass spectrum of air sample in channel 4.

For the demonstration of the mass resolution of each channel, comparative experiments were carried out by using air samples. Figure 5a–d show that all four channels have adequate mass resolution and maintain high consistency within the error tolerance.

## 4. Conclusions

In order to prepare the MFITA with high performance, the internal electric field composition and mass resolution were simulated, by changing structural parameters such as the field radius of exit direction *y*_0_ and ion exit slot width *s*_0_. The simulation results indicate that considering the balance of mass resolution and extraction efficiency, the preferable values for *y*_0_ and *s*_0_ are 1.61 mm and 200 μm, respectively. Combining MEMS and laser etching technology to fabricate MFITA can achieve higher machining accuracy and thinner electrode thickness than traditional machining methods, and reduce the electric field distortion caused by machining errors. Mass spectrometry experiments show that the all four channels of the fabricated miniature ion trap array have adequate mass resolution and maintain high consistency within the error tolerance, which proves the feasibility of fabricating the miniature ion trap array by MEMS technology.

## Data Availability

All data needed to evaluate the conclusions in the paper are present in the paper and/or the Supplementary Materials. Additional data related to this paper may be requested from the authors.

## Supplementary Materials

The following are available online at https://www.mdpi.com/article/10.3390/mi12070831/s1, Note 1: Detailed components and mechanism of the self-built electron impact ion source (EI) mass spectrometer system, Figure S1: (a) The internal electric field distribution of MFITA under a pair of balanced digital bound square wave voltages, (b) The internal electric field distribution of MFITA under a digital excitation square wave voltage, Figure S2: Schematic diagram of mass spectrum, Figure S3: (a) The components of EI mass spectrometer system, (b) Schematic diagram of voltage application mechanism of MFITA.
