# Supplementary material for: A Miniature Four-Channel Ion Trap Array Based on Non-silicon MEMS Technology
Source: Micromachines (Basel). 2021 Jul 16;12(7):831. doi: 10.3390/mi12070831 (PMC8306856; doi:10.3390/mi12070831)
Supplement: Supplementary file 1 [file micromachines-12-00831-s001.zip › micromachines-1297995-supplementary.pdf]

Supplementary Notes

Supplementary Note 1: Detailed components and mechanism of the self-built electron impact ion source (EI) mass spectrometer system.

The test platform used in our experiments is the self-built electron impact ion source (EI) mass spectrometer system, which is comprised of a two-channel high frequency and high voltage square wave generator (HVHF switch300, Shimadzu Research Laboratory (Shanghai) Co. Ltd), a high voltage source (CE 0200 010T, Earthworm Electronics (Shanghai) Co. Ltd), a data acquisition board (CCSA02, Shimadzu Research Laboratory (Shanghai) Co. Ltd), a current-voltage preamplifier (WSA 0002, Shimadzu Research Laboratory (Shanghai) Co. Ltd), a whirlpool dry pump (SVF series, Cisco Vortex Technology Co. Ltd), a molecular pump (HiPace 10, Pfeiffer Vacuum Technology Co. Ltd) and a vacuum chamber that contains an ion source, an ion transmission lens group and our MFITA (Supplementary Figure S3a).

Two signals are generated by the two-channel high frequency and high voltage square wave generator. One is a pair of balanced digital bound square wave voltages (equal amplitude and 180° phase difference) that are applied to adjacent electrode pairs in the x direction, and another is a digital excitation square wave voltage that is also applied to the electrodes in the x direction for the resonance excitation and ejection of ions. The electrodes in the y direction are grounded (Supplementary Figure S3b).

After repeated explorations, a set of optimized parameters used in our mass spectrometry experiments was finally determined. The DC voltages of the repeller, focuser, transmission lens, extraction lens and rear end cap electrode are 15, -88, -3.8, -1 and 15 V, respectively. The front end cap electrode serves as an ion gate, its voltage needs to be lowered during the ion introduction stage to ensure that the ions can efficiently enter the MFITA, the voltage in this stage is 1 V, and in other stages is increased to 20 V. The cooling air is the residual air in the environment, and the pressure is controlled to about 4.5×10^{-4} Torr.

Supplementary Figures

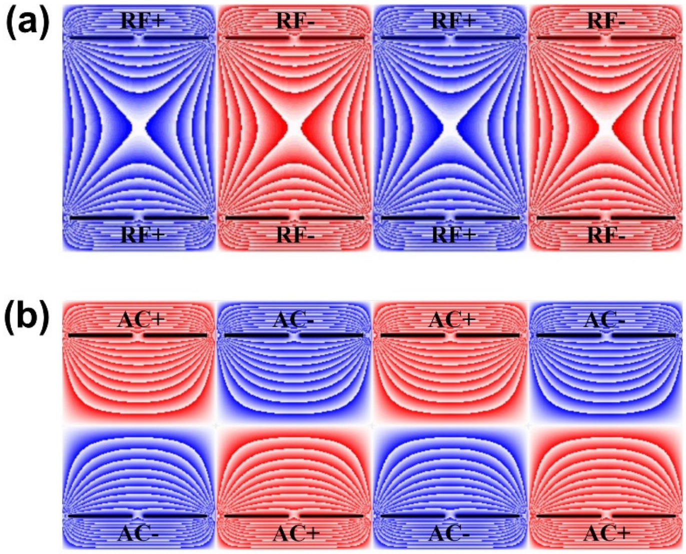

Supplementary Figure S1. (**a**) The internal electric field distribution of MFITA under a pair of balanced digital bound square wave voltages. (**b**) The internal electric field distribution of MFITA under a digital excitation square wave voltage.

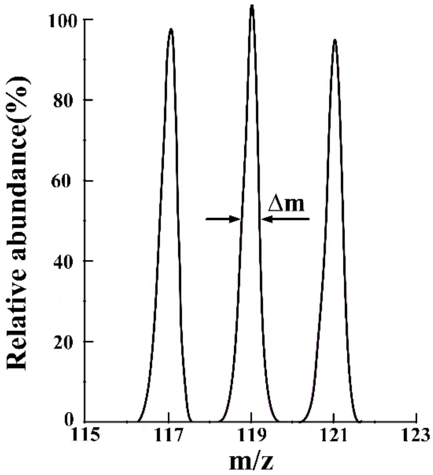

Supplementary Figure S2. Schematic diagram of mass spectrum.

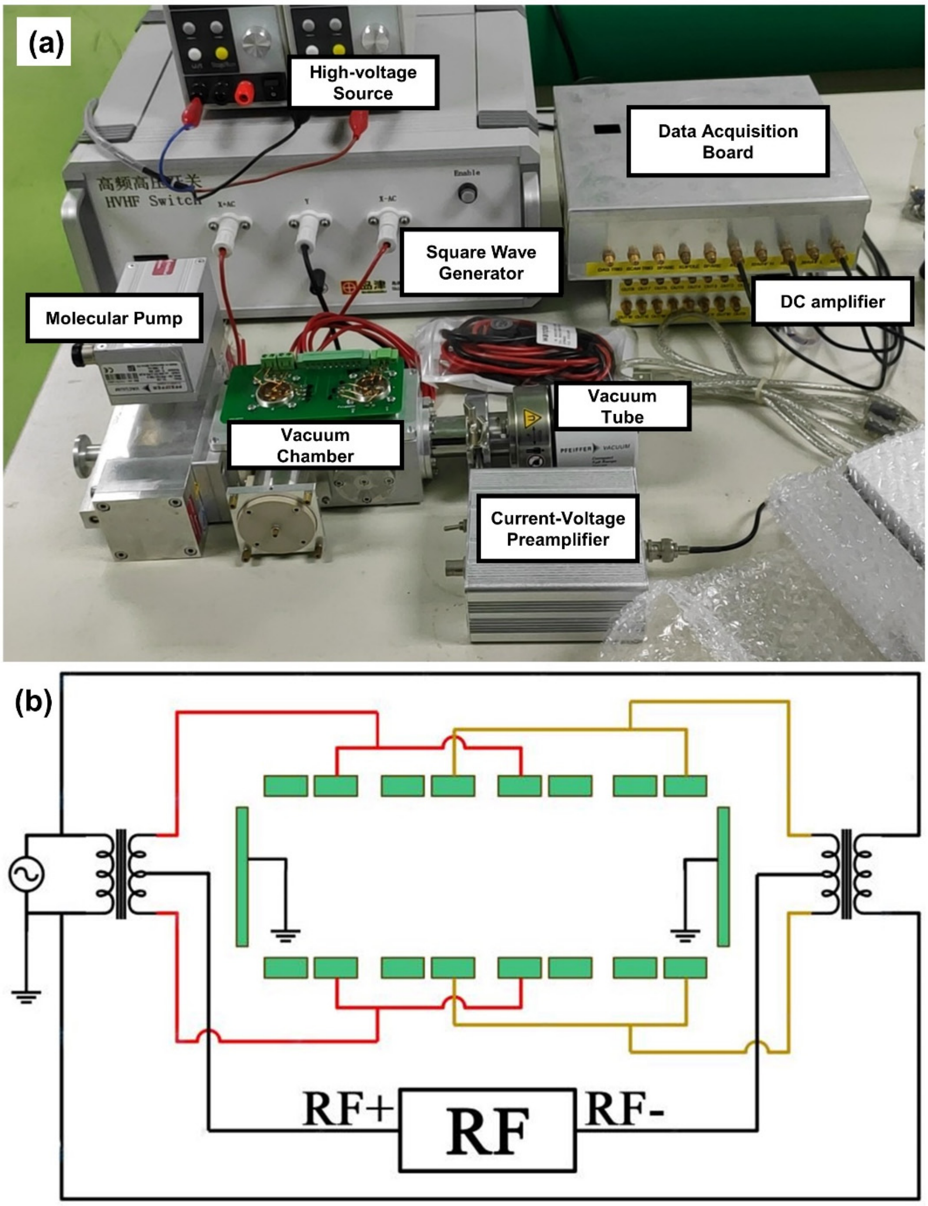

Supplementary Figure S3. (**a**) The components of EI mass spectrometer system. (**b**) Schematic diagram of voltage application mechanism of MFITA.
